# When animal viruses meet N^6^-methyladenosine (m^6^A) modifications: for better or worse?

**DOI:** 10.1186/s13567-024-01424-5

**Published:** 2024-12-18

**Authors:** Wenjing Wang, Yufei Jin, Ziyun Xie, Mei He, Jing Li, Zihan Wang, Saiya Ma, Wuchao Zhang, Jie Tong

**Affiliations:** 1 College of Life Sciences, Hebei University, Baoding, 071002 China; 2 School of Life Sciences and Green Development, Hebei University, Baoding, 071002 China; 3 College of Veterinary Medicine, Hebei Agriculture University, Baoding, 071001 China

**Keywords:** *N*^*6*^-methyladenosine (m^6^a), epigenetic modification, animal virus, virus replication, antiviral immune responses

## Abstract

*N*^*6*^-methyladenosine (m^6^A) is a prevalent and dynamic RNA modification, critical in regulating gene expression. Recent research has shed light on its significance in the life cycle of viruses, especially animal viruses. Depending on the context, these modifications can either enhance or inhibit the replication of viruses. However, research on m^6^A modifications in animal virus genomes and the impact of viral infection on the host cell m^6^A landscape has been hindered due to the difficulty of detecting m^6^A sites at a single-nucleotide level. This article summarises the methods for detecting m^6^A in RNA. It then discusses the progress of research into m^6^A modification within animal viruses’ infections, such as influenza A virus, porcine epidemic diarrhoea virus, porcine reproductive, and respiratory syndrome virus. Finally, the review explores how m^6^A modification affects the following three aspects of the replication of animal RNA viruses: the regulation of viral genomic RNA function, the alteration of the m^6^A landscape in cells after viral infection, and the modulation of antiviral immunity through m^6^A modification. Research on m^6^A modifications in viral RNA sheds light on virus-host interactions at a molecular level. Understanding the impact of m^6^A on viral replication can help identify new targets for antiviral drug development and may uncover novel regulatory pathways that could potentially enhance antiviral immune responses.

## Introduction

RNA modifications play a crucial role in regulating gene expression post-transcription. Among the 170-plus known RNA modifications, N^6^-methyladenosine (m^6^A), 5-methylcytidine (m^5^C), N^1^-methyladenosine (m^1^A), N^1^-methyladenosine (m^1^A), N^4^-acetylcytidine (ac4C), 2′-O-methylated nucleotide (Nm), internal N^7^-methylguanosine (m7G), pseudouridine (Ψ) and A-to-I (inosine) editing have all been reported to participate in virus replication. The reversible nature of RNA modifications allows for precise control of gene expression in response to virus infection, making them essential for anti-viral responses in mammalian cells. Understanding RNA modifications is thus essential for deciphering the complexities of virus infection and the molecular basis of viral-induced diseases. Continued research in this area offers potential to uncover new mechanisms of RNA-based regulation and explore potential therapeutic strategies.

Recent research emphasises the role of m^6^A modifications in viral infections and how they can either promote or inhibit viral replication. N^6^-methyladenine (m^6^A) in mammalian mRNAs was initially discovered in the 1970s [1]. Despite technical limitations, recent advancements in RNA biology have propelled the investigation of RNA m^6^A modification, establishing it as a leading and highly sought-after research area [2, 3]. m^6^A modification is a reversible process orchestrated by a complex interplay of methyltransferases (writers), demethylases (erasers), and proteins that recognise methylated RNA (readers) [4]. Methyltransferases such as METTL3, METTL14, WTAP, METTL16, KIAA1429, RBM15, and ZC3H13 form complexes that carry out the methylation of m^6^A [5]. In contrast, demethylases such as FTO and ALKBH5 remove methyl groups from m^6^A-modified bases [6, 7]. m^6^A readers such as YTHDF1, YTHDF2, and YTHDF3 can identify m^6^A-modified sites and activate the subsequent regulatory processes, including RNA degradation, mRNA translation, and miRNA processing [8, 9]. Indeed, extensive evidence has demonstrated that m^6^A modifications play intricate regulatory roles in nearly all aspects of RNA metabolism [4, 10].

**Figure 1:**
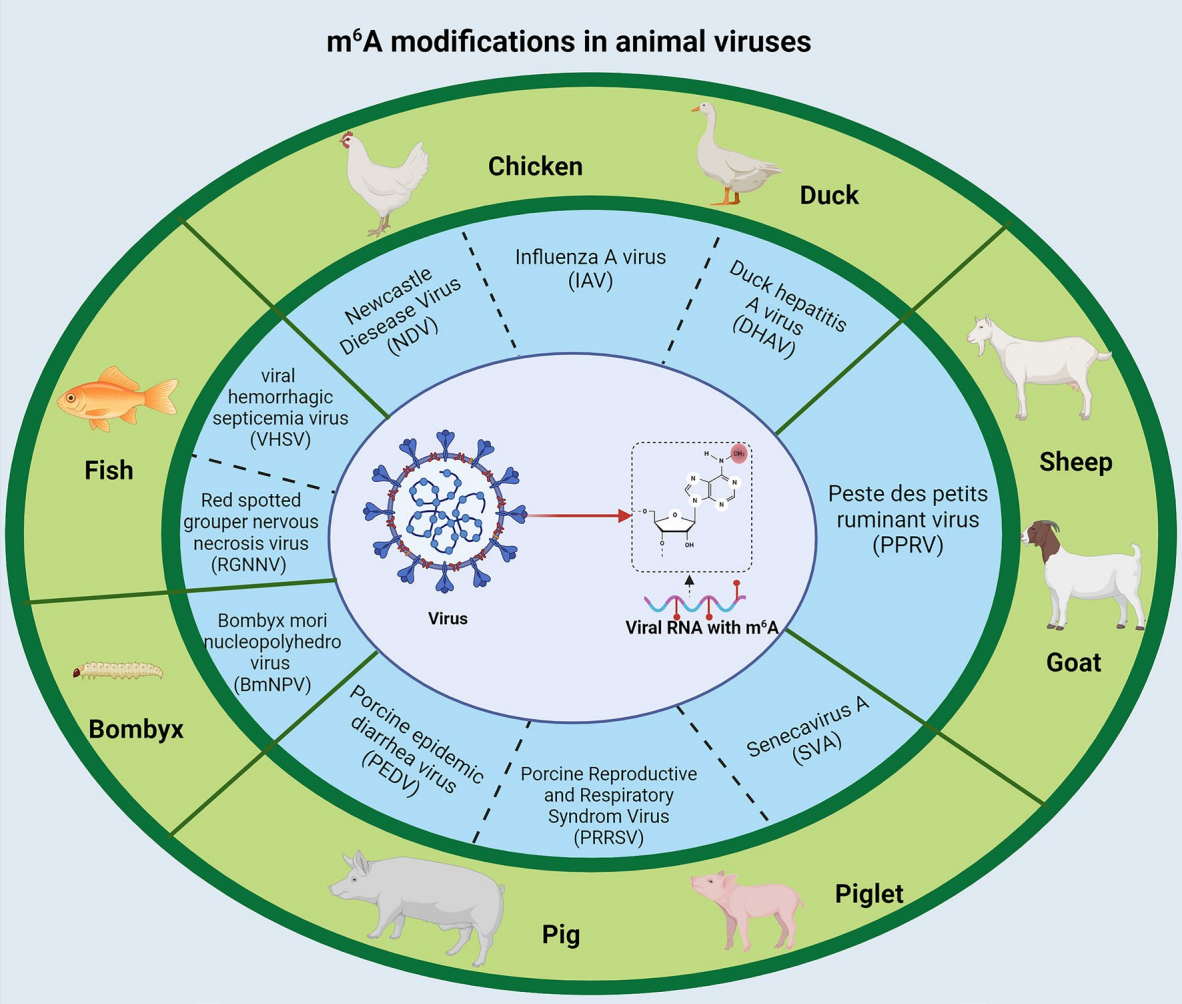
**A brief summary of m**^**6**^**A modifications in animal viruses**. Based on recent publications, it has been discovered that at least nine types of animal viruses, which infect porcine, avian, fish, goat and even bombyx, contain m^6^A modifications in their genomes. Some of these viruses, such as PRRSV and PEDV, are veterinary pathogens that cause significant economic losses worldwide. Therefore, it is crucial to understand the impact of m^6^A on viral replication in order to advance research in animal virology.

Currently, the techniques used to detect m^6^A can be categorised into two groups: those dependent on anti-m^6^A antibodies and those independent of them. For example, high-throughput sequencing techniques combined with liquid chromatography-mass spectrometry (LC–MS) [11], such as meRIP-seq [12] and miCLIP-seq [13–15], enable the detection of m^6^A modifications with high-resolution using anti-m^6^A antibody-dependent methods. Whereas LC–MS/MS methods quantify the total amount of m^6^A in RNAs, similar to colorimetric methods [16]. Notably, based on the biochemical properties of m^6^A, several antibody-free methods have been developed to detect m^6^A at the single-base level. These methods include the high-resolution melting curve method [17] and the SELECT method [18], which both greatly enhance the efficiency and accuracy of m^6^A identification.

m^6^A modifications play a crucial role in regulating mRNA metabolism through various mechanisms. Firstly, m^6^A markedly influences the stability of mRNA [19]. Typically, m^6^A-marked mRNAs have shorter half-lives, but the specific effects also depend on the context and the presence of other RNA-binding proteins [20]. Secondly, the translation efficiency of m^6^A-modified mRNA can be enhanced via the binding of m^6^A reader proteins (e.g. YTHDF1). Additionally, some other m^6^A readers may inhibit the translation efficiency of m^6^A-modified mRNAs [21, 22]. Furthermore, m^6^A regulates pre-mRNA splicing, creating various mRNA isoforms from the same gene loci. m^6^A modification regulates the exporting of mRNA from the nucleus to the cytoplasm, ensuring that mRNA reaches the ribosomes for translation [23, 24]. On the other hand, m^6^A-modified mRNAs can be targeted for degradation through m^6^A-binding proteins such as YTHDF2. These proteins facilitate the mRNAs to cytoplasmic RNA decay sites [25]. Together, these regulatory roles enable m^6^A modifications to finely tune gene expression in response to various cellular signals and environmental conditions.

m^6^A modification plays a significant role in the viral life cycle. It may directly impact the replication and transcription of viral genes. It can also influence the host cell’s anti-viral immune responses by regulating the mRNA metabolism of various antiviral factors [26–28]. As a result, an increasing number of studies have emphasised the specific roles of m^6^A in virus infection [29–32]. Intriguingly, some are specifically dedicated to studying animal viruses [33]. We will systematically summarise the specific impacts of m^6^A modifications on the life cycle of animal viruses, especially RNA viruses (Figure 1). The diverse effects of m^6^A modification on virus replication underscore the intricate and significant role of m^6^A modification in regulating virus-host interactions. This offers a valuable framework for future investigations into the epigenetic regulation of animal virus replication.

## Identification of m^6^A modification in RNA

Adenosine methylation does not change its base-pairing with thymidine or uracil. As a result, m^6^A cannot be easily detected using standard hybridisation or sequencing-based methods. The chemical properties of m^6^A make it resistant to many reagents, which complicates the identification of m^6^A sites at single-nucleotide level. Previously, m^6^A modification sites were primarily identified through RNA–protein interaction combined with high-throughput sequencing [34]. Optically based UV crosslinking co-detection and highly antibody-dependent m^6^A modification site identification methods include meRIP-m^6^A-seq [12], PA-m^6^A-seq [35], miCLIP-m^6^A-seq [36], and so on. Recently, various antibody-independent m^6^A detection methods have been established, including high-resolution melting (HRM) detection [17], m^6^A-REF-seq [37], MAZTER-seq [38], and the BstI DNA polymerase-dependent method [39]. Additionally, electrochemical methods utilising nanopore sequencing technology [40, 41] have also been developed. Table 1 lists the main advantages and disadvantages of m^6^A modification detection methods.

**Table 1:** **Summary of m**^**6**^**A detection methods**

Method	Resolution	Advantages	Disadvantages	Main applications
MeRIP-seq (m^6^A-seq)	Low	Provides a broad overview of m^6^A distribution; widely applicable	Requires antibodies; complex background in results	Transcriptome-wide studies of m^6^A distribution
PA-m^6^A-seq	~30 nts	Low background noise	Multiple complex steps; requires 4-thiouridine and UV crosslinking	Studying m^6^A modifications in viral and cellular RNA, detecting various kinds of RNA modifications
MiCLIP-m^6^A-seq	Single-nucleotide	Highly specific	Complicated procedure; low UV crosslinking efficiency	Precise mapping of m^6^A modifications transcriptome-wide
High-Resolution Melting (HRM)	Single-nucleotide	Simple and quick; antibody-independent	Requires prior knowledge or speculation of m^6^A modification sites	Detecting m^6^A modifications in specific RNA sequences
m^6^A-REF-seq	Single-nucleotide	High-throughput; antibody-independent	Only applicable to specific sequences (ACA)	Precise identification of m^6^A modification sites and methylation levels
MAZTER-seq	Single-nucleotide	Quantitative and high-throughput; antibody-independent	Only applicable to ACA sites; sensitivity and specificity need calibration	Studying m^6^A dynamics in yeast and mammalian systems
BstI DNA Polymerase dependent methods	Single-nucleotide	Simple and quick; antibody-independent	Limited reverse transcription fragment length; requires prior knowledge of m^6^A modification range	Detecting m^6^A modifications in structurally complex RNAs such as viral RNA
Nano-m^6^A	Single-nucleotide	High precision; Direct detection of native RNA	Complex data processing; expensive equipment	Detailed analysis of RNA modifications, including m^6^A and other RNA modifications
ONT Direct RNA Sequencing (DRS)	Single-nucleotide	Preserves native RNA state; no reverse transcription needed; detects multiple RNA modifications	Expensive equipment; complex data processing; requires extensive data analysis	Transcriptome-wide RNA modification detection, including long transcripts and identification of native RNA 3′ ends

### Methyl-RNA immunoprecipitation and sequencing (MeRIP-Seq)

Methyl-RNA immunoprecipitation and sequencing (MeRIP-Seq, also called m^6^A-Seq) relies on an antibody that specifically recognises and binds to the m^6^A site on RNA, enriching the RNA [12]. The RNA transcripts containing m^6^A modification bound to the antibody are then identified by high-throughput sequencing, providing the specific location range of m^6^A modification in RNA. This method is relatively simple and has a wide range of applications. Additionally, different types of RNA modifications, such as N^4^-acetylcytosine (ac4c) [42], can also be detected using different antibodies. However, the results are pretty complex, and the resolution of the detected sequence is relatively low. The RNA fragments obtained through immunoprecipitation may contain m^6^A at various locations [34]. Since the transcripts are fragmented before immunoprecipitation, the m^6^A-seq analysis cannot determine methylation patterns at the single-nucleotide level but instead provides a gene-specific methylation profile [43]. Currently, the MeRIP-seq is the most popular method for mapping m^6^A modification sites in virus-infected cells [44, 45].

### Photo-crosslinked assisted m6A sequencing (PA-m6A-Seq)

PA-m^6^A-seq refers to photo-crosslinked assisted m^6^A sequencing. This method is derived from a widely used protein-RNA interaction assay named photoactivatable ribonucleoside-enhanced crosslinking and immunoprecipitation (PAR-CLIP). It specifically investigates interactions between the m^6^A antibody and RNA [46]. Before RNA extraction, cells of interest are first pulsed with the photoactivatable nucleoside analogue 4-Thiouridine (4SU), which is then incorporated into the transcribed RNA. The extracted 4SU^+^ RNA is then bound to the m^6^A-specific antibody and exposed to UV crosslinking at a wavelength of 365 nm. This efficiently crosslinks 4SU^+^ residues to RNA-bound proteins. The process involves collecting crosslinked antibody-RNA complexes through immunoprecipitation and releasing RNA fragments using proteinase K digestion. This causes amino acid residuals to bind to the previously crosslinked 4SU. This leads to misincorporation in the reverse transcription step in preparation for RNA-seq, resulting in a T > C mutation at the cross-linked site [35]. This method has a high resolution of 32 nts. Additionally, after ribonuclease treatment, the RNA that is not bound to and protected by antibodies is digested, reducing the background for RNA-seq. However, this method is relatively more gradual and complex due to the multiple procedures [47]. It has been used to detect various RNA modification sites in viral ribonucleic acid transcripts in viruses [48–51].

### m6A individual-nucleotide-resolution crosslinking and immunoprecipitation (miCLIP)

meRIP-m^6^A-seq and PA-m^6^A-seq methods only determine the existence of m^6^A modification in a segment of RNA, while the miCLIP method determines the presence of m^6^A modification sites at the single base level. The use of ultraviolet light to cross-link antibodies with RNA depends on the interaction between RNA and m^6^A-specific antibodies [36]. Reverse transcription of crosslinked RNA then produces a highly specific pattern of mutations or truncations in the cDNA. These mutation patterns are then identified using computational methods to pinpoint the exact positions of m^6^A residues [13]. However, the complexity of this method and the low efficiency of UV crosslinking may lead to some m^6^A residues being missed during the identification process.

### High-resolution melting (HRM) analysis

A high-resolution melting (HRM) analysis method has been proposed based on the thermal stability of m^6^A residues in RNA. This method is simple and easy but requires knowing the accurate sequence before identification. The process involves hybridising the RNA with two fluorescent probes and obtaining high-resolution melting curves by monitoring fluorescence. An unmodified control with the same sequence as the samples to be measured is required for comparisons. A lower melting temperature, compared to the unmodified control, indicates the presence of m^6^A at the specific site [17]. While this method has only been used to detect m^6^A sites in rRNA and snoRNA of eukaryotes, it may have advantages in analysing m^6^A modification in viral genome and viral-infected cells.

### m6A-sensitive RNA-endoribonuclease-facilitated sequencing (m6A-REF-seq)

m6A-REF-seq is a precise and high-throughput antibody-independent m6A identification method known as the m6A-sensitive RNA endoribonuclease-facilitated sequencing method. It is based on the m6A-sensitive RNA endoribonuclease, which recognises and cleaves ACA but not the m6ACA motif. This method enables the identification of transcriptomic m^6^A sites and the quantification of methylation levels at single-base resolution [37]. For instance, MazF and ChpBK, both part of the bacterial toxin-antitoxin system, were able to differentiate m^6^A from unmethylated A. MazF recognised and cleaved the motif sequence ACA at the first A, leaving the methylated m^6^ACA motif. Similarly, the cleavage activity of ChpBK was also inhibited by m^6^A at the recognition motif UAC. MazF or ChpBK first digests the measured RNA. If there is no m^6^A modification, the reverse transcription reaction will stop at the A position of the ACA sequence due to the digestion by MazF of ChpBK. However, if there is an m^6^A modification, the reverse transcription reaction will not stop, and the full-length gene will be amplified and sequenced in the following procedure. It is important to note that this method is only suitable for identifying m^6^A modification sites in ACA motifs. It has been used to track m^6^A dynamics in yeast and mammalian systems, as well as to investigate m^6^A function [38].

### Bst I DNA polymerase-dependent m6A detection

Some studies have found that BstI DNA polymerase has reverse transcriptase activity that is even higher than that of AMV reverse transcriptase in some cases. However, the cDNA length is limited to less than 200 nts. It was discovered that the reverse transcriptional activity of BstI DNA polymerase was affected by m^6^A modification. When m^6^A is present in the RNA template, it hinders cDNA synthesis during BstI-catalysed reverse transcription. When there is m^6^A modification in the RNA template, the twist angle will be significantly changed, resulting in the loss of the conformational flexibility necessary for BstI progression. As a result, a combination of BstI-catalysed reverse transcription and primer extension or RT-PCR/qPCR can be used to identify m^6^A modifications in RNA [39, 52, 53]. Furthermore, BstI has a working temperature of up to 65 °C, demonstrating higher thermal stability. Therefore, using BstI in reverse transcription can minimise the influence of RNA secondary structures, making the method more effective for detecting RNA with complex secondary structures, such as viral RNA genomes.

### Other electrochemical methods

The nano-m^6^A detection pipeline uses nanopore-direct RNA sequencing and an extreme gradient boosting model to identify and quantify m6A modifications at a single-base resolution accurately. This approach allows for the precise detection and visualisation of m^6^A sites within individual transcripts by leveraging the raw signal surrounding these sites [54]. Oxford Nanopore Technologies (ONT) long-read sequencing is particularly efficient in identifying m^6^A sites [55]. When a single-stranded RNA passes through a membrane in a nanopore with a voltage, the nucleotides present in the pore will affect the pore’s electrical resistance, yielding many possible states: 45 = 1024 for a standard four-base model. If modified bases are present in the RNA sample, such as 5-methylcytosine, the number of possible states can increase further: 55 = 3125 [56]. This electrical signal is the raw data gathered by an ONT sequencer. The benefits of ONT technology include the direct sequencing of natural RNA (direct RNA sequencing, or DRS). This method can accurately represent the state of RNA after transcription and preserve information about RNA modifications [56]. DRS allows for detecting long transcripts that may not be identified by short reading length technology, and it can pinpoint the original 3’terminal sites of natural RNA [57, 58]. Additionally, the Nanpolish [59] and DeepSignal [60] methods can identify various types of precise modification sites at the single-molecule level, such as 5-methylcytosine (m^5^C) [61], N^7^-methylguanosine (m^7^G) and A-to-I editing [62]. Despite its advantages, there are limitations to using DRS technology to detect m^6^A modifications. DRS may struggle to detect m^6^A modifications in low abundance [62] accurately. The resolution of DRS might not be sufficient to confidently distinguish between modified and unmodified nucleotides, leading to false negatives or imprecise quantification [63]. Additionally, the sequencing process can be influenced by the secondary and tertiary structures of RNA molecules, potentially masking or mimicking the effects of RNA modifications and resulting in inaccurate detection or high error rates [64]. Moreover, the DRS method requires a large amount of starting material, which makes it relatively low throughput and expensive compared to other sequencing technologies [65]. This limits its usefulness for large-scale studies, particularly when screening for multiple RNA modifications across a large number of samples. While the ONT method has been used to detect m^6^A sites in HIV mRNA [66], its applications in detecting m^6^A sites in animal viruses are still unclear.

## The diverse roles of m^6^A in regulating viral replication

Due to the extensive impacts of m^6^A modifications on mRNA metabolism, when viruses invade host cells, m^6^A modifications in the viral genomic RNA can regulate viral replication in various ways. This can either facilitate or inhibit virus infection. Typically, when m^6^A modifications enhance the translation of viral RNA, they promote viral replication. Conversely, if m^6^A modifications lead to the degradation of viral RNA, then m^6^A would have a negative regulatory effect on viral infection. Additionally, viral infection may alter the m^6^A landscapes of host cell transcripts, ultimately regulating the anti-viral or pro-viral responses within the host cells.

### The positive roles of m^6^A modifications in virus infection

m^6^A modifications have been reported to play positive roles in replicating several animal viruses, including influenza A virus (IAV), peste des petits ruminant virus (PPRV), and PEDV.

IAV is an RNA virus that can infect many hosts, including humans, avians, and livestock. A study found that overexpression of YTHDF1 and YTHDF2 in IAV-infected cells led to a significant increase in viral encoding genes such as NS1, NP, and M2. This led to an increase in viral titre from 5.5 × 10^6^ PFU/mL to 5.3 × 10^7^ PFU/mL compared to wild-type cells. Conversely, the suppression of YTHDC1, YTHDF2, or METTL3 in IAV-infected cells led to a decrease in viral titre from 10^4^ TCID_50_/mL to 10^3^ TCID_50_/mL at 24 h post-infection (hpi). This suggests that m^6^A modification in the IAV genome positively regulates virus replication by enhancing the translation of viral genes.

PPRV frequently infects goats (*Capra hircus*) and sheep (*Ovis aries*), leading to acute pneumonia or severe diarrhoea. Vero cells were pre-treated with m^6^A inhibitors (3-DAA) and then infected with PPRV. Compared with the untreated group, the expression of PPRV nucleocapsid protein decreased from 10^6^ TCID_50_/mL to 10^3^ TCID_50_/mL. This indicates that the m^6^A inhibitors suppressed PPRV replication in Vero cells. Additionally, reducing METTL3 led to a significant decrease in PPRV N protein mRNA levels after 72 hpi compared to naïve cells. The virus titre also dropped from 10^6^ TCID_50_/mL to 10^4^ TCID_50_/mL. Meanwhile, deleting FTO increased the mRNA levels of PPRV N protein [67]. In conclusion, the replication of PPRV in Vero cells is influenced by m^6^A methyltransferase (METTL3) and demethylase (FTO), indicating a positive role of m^6^A modification in regulating PPRV replication.

Studies have identified m^6^A modifications in the genomic RNA of PEDV by using meRIP-seq. High levels of m^6^A modifications were found in the non-structural protein genes, such as Nsp9-13 and Nsp16, as well as the 3’ untranslated region. However, the specific m^6^A sites within the PEDV genome remain unclear [68]. A recent study found that hnRNP (a type of m^6^A reader) downregulated the expression of TRAF3 through the METTL3-METTL14-YTHDF2 axis. This, in turn, suppressed the expression of IFN-β and the downstream antiviral genes in cells infected with PEDV. In a further study, it was observed that hnRNP was overexpressed during PEDV (MOI = 0.5) infection. After 24 h post-infection, cells and supernatant were collected to analyse the expression of PEDV N protein and virus titre. The virus titre increased from 10^3^ TCID_50_/mL to 10^5^ TCID_50_/mL compared to mock cells. Similarly, following the transfection of cells with anti-hnRNPU siRNA (but not normal control (NC) siRNA), the virus titre decreased from 10^4^ TCID_50_/mL to 10^3^ TCID_50_/mL at 24 h post-PEDV infection. The overexpression of m^6^A reader has thus been found to promote PEDV replication. Another study showed that overexpression of m^6^A eraser ALKBH5 restricted PEDV infection [69]. Additionally, transfection with siMETTL3 or siYTHDF2 reduced the mRNA levels of PEDV N protein from 1.0 to 0.5 and 0.3, while siMETTL14 had no significant effect on the expression of PEDV N protein. Both YTHDF2 and METTL3 were found to promote PEDV replication [70]. In summary, m^6^A modification in the PEDV genome may promote viral replication.

### The negative roles of m6A modifications in virus infection

Understanding the molecular mechanisms underlying antiviral responses in fish is crucial for aquaculture health management. LjMETTL3 is a METTL3 homolog gene that was cloned from a sea perch. Notably, LjMETTL3 was more abundant in the immune tissues of sea perch post-red spotted grouper nervous necrosis virus (RGNNV) or viral haemorrhagic septicaemia virus (VHSV) infection. Furthermore, LjMETTL3 expression was significantly upregulated at 12 h and 24 h post-RGNNV and VHSV infection in-vitro. In addition, ectopic expression of LjMETTL3 inhibited RGNNV and VHSV infection in LJB cells, whereas the knockdown of LjMETTL3 led to opposite effects [71]. The higher levels and increased activity of LjMETTL3 in response to RGNNV and VHSV infections indicate its essential role in the sea perch’s immune response to viruses. The ability of LjMETTL3 to inhibit these viral infections highlights its potential as a target for improving viral resistance in aquaculture.

Among the viral genes associated with the replication and proliferation of bombyx mori nucleopolyhedrovirus (BmNPV), the ie-1 mRNA exhibited a notably higher m6A level compared to other viral genes [72]. The presence of m6A sites in the ie-1 mRNA seemed inversely related to protein expression. When BmYTHDF3 was overexpressed, it led to a dose-dependent inhibition of viral replication. On the other hand, cells transfected with siRNA targeting BmYTHDF3 showed a contrasting effect. Depletion of BmYTHDF3 in BmN cells resulted in increased expression of the viral structural protein VP39, while overexpression of m6A-related enzymes in BmN cells had the opposite effect [73]. The findings highlight the significant role of m^6^A modifications and m^6^A-binding proteins in regulating BmNPV replication. The inverse relationship between m^6^A levels on ie-1 mRNA and protein expression suggests a mechanism in which m^6^A negatively impacts viral gene expression. BmYTHDF3 seems to play a crucial role in this regulatory pathway. Therefore, m^6^A modifications are a negative regulatory mechanism for BmNPV replication in BmN cells. Targeting m^6^A-related enzymes could offer new strategies for controlling BmNPV infections.

### m^6^A modification and regulation of viral-host interactions

High-pathogenicity porcine reproductive and respiratory syndrome virus (HP-PRRSV) is a virus that infects farmed pigs worldwide. HP-PRRSV causes significant changes in m^6^A modification levels and alternative splicing of many genes in lung tissue, including LMO7, SLC25A27, ZNF185, and ECM1 [74]. These genes play crucial roles in cellular metabolism. For example, ECM1 may regulate viral extracellular spread via the ITGB3-AKT2/FAK signalling pathway, while LMO7 may modulate inflammatory signalling pathways by inhibiting the expression of genes such as c-JUN and SMAD3. Recent studies have shown that the HP-PRRSV Nsp9 antagonises FTO expression. This action leads to an increase in endogenous mRNA m^6^A modification levels and affects the expression of crucial host factors such as IL-13 [75]. Therefore, changes in the m^6^A modification landscape may play a key role in virus-host interactions during PRRSV infection.

The Newcastle disease virus (NDV) significantly impacts poultry industries globally. Understanding the interactions between the virus and its host at a molecular level is crucial to developing effective strategies to combat it. One such interaction involves m^6^A modifications, critical in regulating gene expression [76]. Following infection with NDV, 1234 mRNAs showed significantly altered m^6^A methylation levels. A negative correlation was found between m^6^A modification and cellular mRNA expression, suggesting that m^6^A may suppress the expression of specific host genes during infection. The study found that many affected mRNAs play a role in the innate immune response, suggesting that m^6^A modifications may affect the host's defence mechanisms against NDV infection. The infection also caused an increase in m^6^A-related enzymes, potentially altering the m^6^A landscape in the host cell's transcriptome. Furthermore, m^6^A peaks were identified in the NDV genome, indicating that host methylation-related enzymes could influence viral replication [77].

Senecavirus A (SVA) is a significant veterinary pathogen with a single-stranded mRNA genome. Epigenetic modifications, like m^6^A, are known to affect the replication and evolution of SVA. While the SVA genome may undergo m^6^A modification during replication, the analysis showed that only half of the viral genomic RNA samples (three out of six) exhibited m^6^A modifications [78]. The presence of m^6^A modifications varies across samples, suggesting that these epigenetic effects may not significantly drive SVA evolution. Further research is needed to explore alternative epigenetic regulatory mechanisms influencing SVA replication and adaptation.

The enrichment of m^6^A modification in the host cell’s transcriptome may be altered by virus infection. It has recently been shown that the m^6^A levels in endogenous mRNA were significantly higher in the livers of ducks infected with the attenuated duck hepatitis A virus (DHAV) compared to those infected with high-virulent strains [79]. The combined analysis of m^6^A-RIP-seq and RNA-seq showed a generally positive correlation between m^6^A modification levels and mRNA expression levels in the livers of ducklings infected with DHAV. The increased m^6^A levels in attenuated DHAV-infected livers suggest a potential role for m^6^A in modulating the host's response to viral infection. The positive correlation between m^6^A modification and mRNA expression suggests that m^6^A may improve the stability and translation of host mRNAs, thereby influencing the outcome of DHAV infection. As a result, m^6^A modification may play a crucial role in DHAV infection, especially in attenuated versus highly virulent strains.

Moreover, although some animal viruses have been reported to carry m^6^A modifications in their genome, the exact functions of m^6^A modifications in viral life cycles remain unclear. For instance, the avian leukosis virus subgroup J (ALV-J) [33].

## How does m^6^A modification regulate virus replication?

### m^6^A modification in viral genomes and regulation of viral RNA metabolism

**Figure 2:**
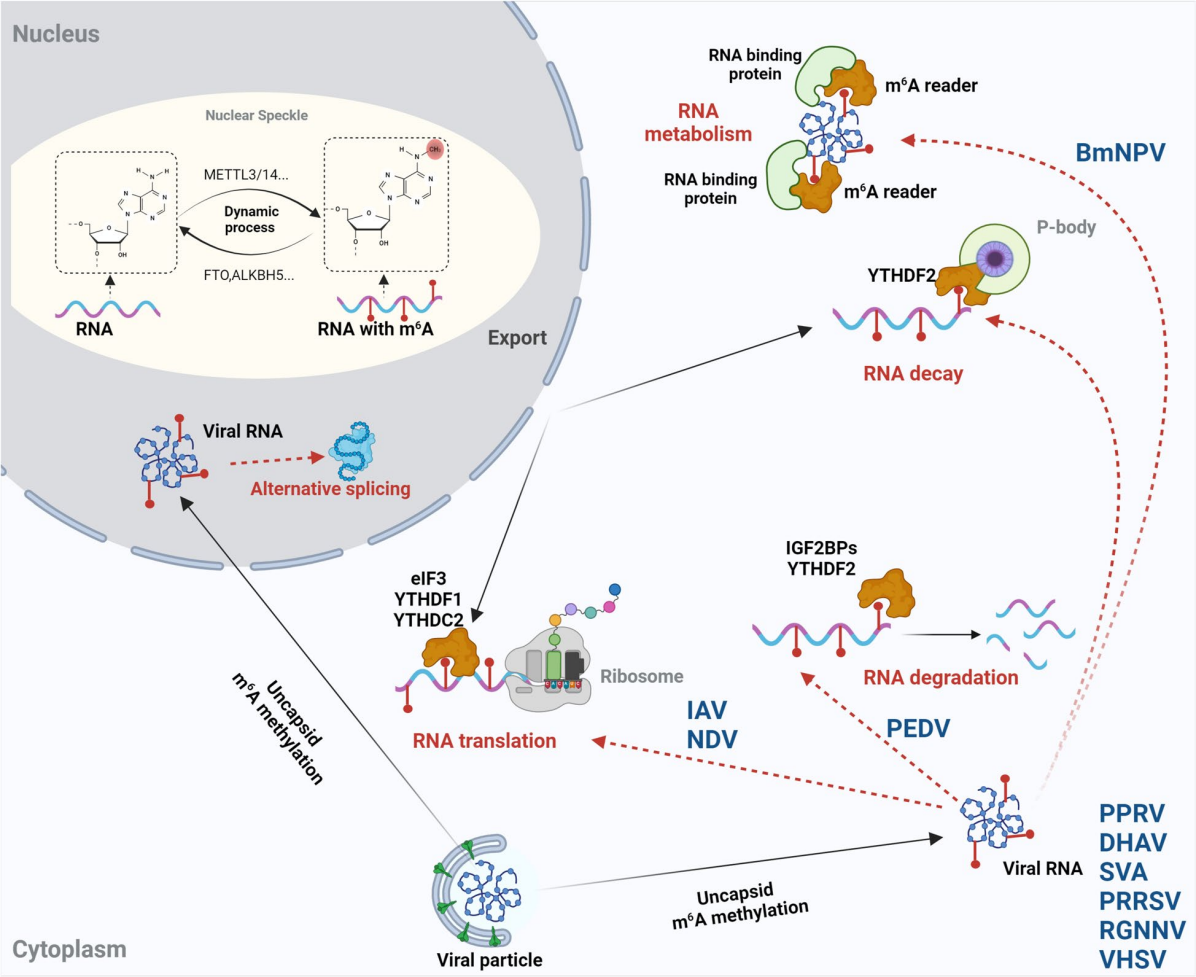
**m**^**6**^**A modification regulates viral RNA metabolism**. The presence of m^6^A modifications within viral genomes can significantly impact viral replication by affecting viral RNA functions. These modifications influence viral gene expression by impacting the stability, splicing, and translation of viral RNA. m^6^A reader proteins, such as YTHDF and IGF2BP, recognise the modified viral RNA and facilitate its metabolism in the cytoplasm. For example, the YTHDF1 and YTHDC2 promote the translation of m^6^A modified viral RNA, while YTHDF2 recruits other host cell factors to degrade m^6^A modified viral RNA. These opposing effects highlight the complexity of m^6^A-mediated regulation of viral genomes and emphasise the need for further investigation to fully understand how m^6^A modifications influence viral replication.

The presence of m^6^A modifications within viral genomes can significantly affect viral replication by changing viral RNA functions. These modifications impact viral gene expression by influencing viral RNA's stability, splicing, and translation (Figure 2). For example, m^6^A modifications have been found in the HA genes of IAV. These modifications increase the HA protein's expression by improving HA mRNA's stability [80]. In other viruses, such as HIV and ZIKV, m^6^A modifications help in the replication of the virus by increasing the stability and translation of viral mRNA [32, 81]. Specifically, the methylation of viral RNA by the host methyltransferase complex, which consists of METTL3 and METTL14, plays a crucial role in stabilising viral RNA and facilitating efficient translation. This promotes viral gene expression and replication. On the other hand, in different contexts, m^6^A modifications can act as a repressive mechanism. For instance, m^6^A modifications have been discovered to decrease the stability of HBV pgRNA, thereby inhibiting viral replication [82–84]. These conflicting effects emphasise the complexity of m^6^A-mediated regulation of viral genomes and underscore the need for further investigation to fully understand the mechanisms by which m^6^A modifications influence viral replication.

### Affecting m^6^A levels of cellular transcriptome post-viral infection

Virus infections significantly change the host cell's transcriptome, including modifications in the m^6^A landscape of cellular RNAs. These changes can impact the expression of host genes crucial for the viral life cycle and the host's antiviral response. For example, during IAV infection, there is a widespread increase in m^6^A modifications on host mRNAs [80]. This alteration affects the stability and translation of host mRNAs, potentially facilitating viral replication or enhancing the host's antiviral defence mechanisms.

In the case of human cytomegalovirus (HCMV) infection, m^6^A modifications on host mRNAs are dynamically regulated [85]. HCMV induces the expression of m^6^A writer enzymes such as METTL3 and METTL14, which modify specific host mRNAs to favour viral replication. This virus-induced reprogramming of the host m^6^A landscape can lead to the upregulation of pro-viral genes and the downregulation of antiviral genes, thereby creating a more favourable cellular environment for viral replication.

Furthermore, m^6^A modifications can influence the expression of key regulatory genes involved in virus-host interactions. For instance, m^6^A modification in the interferon-stimulated gene 15 (ISG15) mRNA can modulate its expression, impacting the antiviral response [86, 87]. Another potential target of m^6^A modifications in regulating anti-viral responses is the tripartite motif containing 29 (TRIM29). TRIM29 has been shown to regulate both DNA and RNA virus infections by mediating type I interferon (IFN-I) responses [88, 89]. For example, the Epstein-Barr virus (EBV) has been reported to use TRIM29 to suppress innate immune responses in host cells, leading to persistent infections [90].

TRIM29 is also capable of directly binding to NEMO and subsequently inducing its ubiquitination and proteolytic degradation in response to influenza virus and *Haemophilus influenzae* virus infections in the respiratory tract [88]. Additionally, TRIM29 enhances PERK-mediated ER stress immune responses, thereby promoting viral myocarditis induced by cardiotropic RNA viruses [91].

Recent research has indicated that recruitment of YTHDF1 to m^6^A-modified TRIM29 is involved in promoting TRIM29 translation in cisplatin-resistant ovarian cancer cells [92]. Therefore, TRIM29 may be a crucial mediator in regulating virus-host interactions via m^6^A modifications. These findings suggest that viruses can manipulate the host m^6^A machinery to evade immune responses and enhance their replication.

### Regulating antiviral immunity through m^6^A modifications

**Figure 3:**
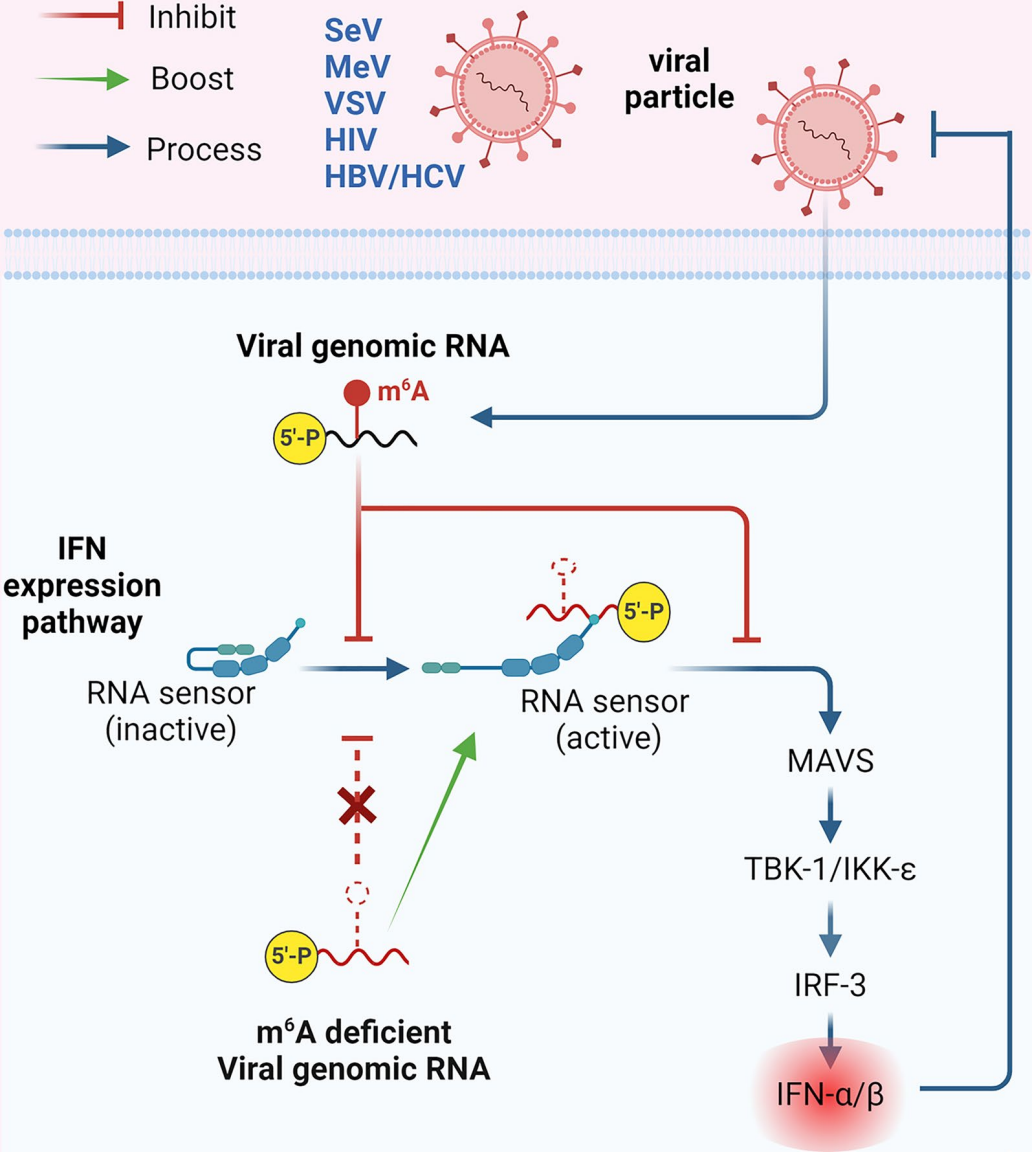
**m**^**6**^**A modification regulates IFN production**. The activation of pattern recognition receptors (PRRs) that recognise viral RNA is influenced by m^6^A modifications. This modulates the innate immune response. Additionally, the signalling transduction by MAVS, the adaptor of RNA sensors, is also influenced by m^6^A modifications in viral RNA. As a result, the production of IFN-I is affected, which further regulates the antiviral innate immune response in virally infected cells.

m^6^A modifications are crucial in regulating the body’s antiviral immune response, including innate and adaptive immunity. The innate immune response is the first line of defence against viral infections. m^6^A modifications influence the activation of pattern recognition receptors (PRRs) that recognise viral RNA, thereby affecting the innate immune response (Figure 3). For instance, m^6^A-modified viral RNA can hinder recognition by the cytoplasmic RNA sensor RIG-I, enabling the virus to evade the activation of downstream signaling pathways and the production of type I interferons (IFNs) [28]. This diminishes the antiviral state of the host cell and hinders enhanced virus replication. Furthermore, m^6^A modifications can impact the expression of key regulators of the innate immune response, such as interferon regulatory factors (IRFs) and nuclear factor-kappa B (NF-κB), thus influencing the strength and duration of the antiviral response [87].

During adaptive immune responses, m^6^A modifications can influence the differentiation and function of T and B lymphocytes, which are critical for clearing viral infections. For example, m^6^A modifications on mRNAs encoding cytokines and chemokines can regulate their expression, shaping the immune response [93]. Targeting the m^6^A machinery in immune cells can enhance the efficacy of antiviral therapies by boosting the host's immune response against the virus.

## Conclusions and future prospects

The regulation of viral replication by m^6^A modifications is a complex process that involves modulating viral genome RNA functions, changes in cellular m^6^A landscapes, and the regulation of antiviral immunity. Understanding these interactions provides valuable insights into the molecular mechanisms of viral replication and virus-host interactions. Targeting m^6^A modifications shows promise for developing novel antiviral therapies. By modulating the m^6^A machinery, it may be possible to enhance antiviral immune responses and inhibit viral replication, offering new strategies to combat viral infections.

However, targeting the m^6^A machinery for therapeutic purposes presents a complex challenge due to its integral role in cellular gene expression. It requires nuanced approaches to selectively target viral components without disrupting cellular functions. One possible approach is to develop small molecules or engineered proteins that can specifically recognise and bind to m^6^A-modified sites on viral RNA without affecting cellular RNA. This could be achieved by identifying unique sequences or structural features in viral RNA that are absent in cellular RNA.

Targeting the interaction between viruses and their hosts could help reduce side effects. Some viruses use specific host proteins to alter their RNA. Disrupting these interactions might selectively prevent the m^6^A modification of viral RNA without affecting cellular RNA. However, this approach requires precise control over the timing and dosage of therapeutic agents to reduce viral RNA modifications during critical stages of the viral life cycle while allowing normal cellular processes to proceed.

Significant progress in RNA biology has provided precise methods for interfering with the m6A modification in viral RNA in recent years. For instance, the CRISPR/Cas-based systems, like CRISPR/dCas9 [94–96], can be fused to m^6^A writers or erasers and engineered to target viral RNA sequences selectively. By designing guide RNAs that specifically recognise viral RNA, it may be possible to modulate m^6^A marks on viral RNA with high precision. Furthermore, RNA aptamers or nanobodies that specifically bind m^6^A-modified viral RNA could be developed to block the function of these modified sites, inhibiting viral replication without interfering with cellular m^6^A-modified RNA.

Apart from m^6^A modifications, other types of RNA modifications, such as m^1^A and m^5^C, have also been found to play crucial roles in viral replication. The discovery of these modifications in the genomes of RNA viruses suggests that epigenetic marks are not just passive bystanders but active participants in the viral life cycle. They impact various stages of viral replication, including RNA stability, translation, and the evasion of host immune responses. For example, m^1^A modifications have been demonstrated to affect RNA secondary structure and translation efficiency, potentially impacting viral protein synthesis. Similarly, m^5^C modifications can affect RNA stability and the recognition of viral RNA by the host's immune system.

RNA modifications have implications beyond just viral replication. They also affect virus-host interactions and viral evolution. Epigenetic modifications can alter the host's response to viral infections, influencing the infection’s outcome [97]. RNA modifications are dynamic, allowing viruses to adapt quickly to changing host environments, contributing to their evolutionary success. This adaptability is critical in the context of emerging viral pathogens, where rapid evolution can lead to the emergence of new strains with altered pathogenicity and transmissibility.

In conclusion, the study of RNA modifications in viral genomes is a growing field with the potential to enhance our understanding of virus-host interactions and viral evolution. By uncovering the roles of different RNA modifications, such as m^6^A, researchers can discover new mechanisms of viral replication and identify potential targets for antiviral therapies. Exploring epigenetic modifications in viral RNA is an important area for future research, with significant implications for virology and the control of infectious diseases.

## Data Availability

Data availability statement:The data that support the conclusions of this review are available from the corresponding author J.T, upon reasonable request.
